# Using the Gravity Model to Estimate the Spatial Spread of Vector-Borne Diseases 

**DOI:** 10.3390/ijerph9124346

**Published:** 2012-11-30

**Authors:** José Miguel Barrios, Willem W. Verstraeten, Piet Maes, Jean-Marie Aerts, Jamshid Farifteh, Pol Coppin

**Affiliations:** 1 Biosystems Department M3-BIORES, KU Leuven, Willem de Croylaan 34 B3001, Heverlee, Belgium; Email: jean-marie.aerts@biw.kuleuven.be (J.-M.A.); jamshid.farifteh@biw.kuleuven.be (J.F.); pol.coppin@biw.kuleuven.be (P.C.); 2 Climate Observations, Royal Netherlands Meteorological Institute, PO Box 201 NL-3730 AE, De Bilt, The Netherlands; Email: w.w.verstraeten@tue.nl; 3 Applied Physics, Eindhoven University of Technology, PO Box 513 5600 MB, Eindhoven, The Netherlands; 4 Laboratory of Clinical Virology, National Reference Laboratory for Hantaviruses, KU Leuven, Minderbroedersstraat 10 B3000, Leuven, Belgium; Email: piet.maes@rega.kuleuven.be

**Keywords:** gravity models, nephropathia epidemica, Lyme borreliosis

## Abstract

The gravity models are commonly used spatial interaction models. They have been widely applied in a large set of domains dealing with interactions amongst spatial entities. The spread of vector-borne diseases is also related to the intensity of interaction between spatial entities, namely, the physical habitat of pathogens’ vectors and/or hosts, and urban areas, thus humans. This study implements the concept behind gravity models in the spatial spread of two vector-borne diseases, nephropathia epidemica and Lyme borreliosis, based on current knowledge on the transmission mechanism of these diseases. Two sources of information on vegetated systems were tested: the CORINE land cover map and MODIS NDVI. The size of vegetated areas near urban centers and a local indicator of occupation-related exposure were found significant predictors of disease risk. Both the land cover map and the space-borne dataset were suited yet not equivalent input sources to locate and measure vegetated areas of importance for disease spread. The overall results point at the compatibility of the gravity model concept and the spatial spread of vector-borne diseases.

## 1. Introduction

The observed spatio-temporal patterns of vector-borne diseases (VBD) are the response to a wide variety of factors, notably those affecting the ecology of vectors, hosts and pathogens, impacting their demography and the species balance of ecosystems and shaping human exposure to pathogens. 

The spatial nature of VBD risk factors has encouraged the use and development of spatial analysis tools to deepen the understanding of the underlying mechanisms of disease spread, to elaborate prevention and/or action plans and to detect changes in spatial spread of disease. The latter aspect is one of special relevance as climate change may induce expansion and/or translocation of disease epidemic foci. In this respect, both, vector/host habitats and human settlements can be conceived as spatial entities interacting with one another in the context of a given landscape configuration. Hence, spatial notions of common use in landscape ecology like distance, contiguity, cover fraction, *etc*. are applicable in disease modelling when the focus is laid on ecosystems hosting disease vectors and their relation to human settlements. 

Therefore, modelling the spatial spread of VBD where the involved organisms have a well-defined habitat should be supported by existing techniques of spatial interaction analysis. This study presents the implementation of a spatial interaction model that aims at relating VBD risk to the location of vector/host habitat with respect to urban areas. The chosen modelling framework is the gravity model (GM) which, despite being the most widely used spatial interaction model [1], has not been tested in modelling VBD. 

The implementation of the GM was made on the spatial spread of nephropathia epidemica (NE) and Lyme borreliosis (LB) in Belgium. Both diseases have gained much attention in recent years as abnormally high incidences have been reported in Belgium and other European countries [2,3]. The causal agent of NE is the *Puumala virus* (PUUV) hosted by the bank vole *Myodes glareolus*. LB is caused by the bacterium *Borrelia burgdorferi*, transmitted to humans during blood meals of the tick *Ixodes ricinus*. 

Based on current knowledge on NE and LB transmission mechanisms, this study aims at testing the suitability of the GM concept in modelling the spatial spread of these vector-borne diseases. The study responds to the growing need of innovating methods and datasets in VDB surveillance in a period of marked alterations in the usual pattern of VBD [4]. 

## 2. NE and LB in Belgium

LB and NE are zoonotic diseases resulting from two different but related transmission mechanisms. The bacterium *Borrelia burgdorferi* is the causal agent of LB and *Puumala virus* (PUUV) causes NE, a mild form of haemorrhagic fever with renal syndrome. The pathways of both pathogens converge in the vegetative system that hosts their reservoirs. 

The specific vector of PUUV in Western Europe, the bank vole (*Myodes glareolus*), is a native rodent species in temperate forests. *B. burgdorferi* is transmitted to humans by means of bites by ticks of the genus *Ixodes*. Besides its prominent role in the transmission of PUUV, the bank vole is known to be an important reservoir in the transmission chain of *B. burgdorferi*, especially during the ticks’ larval and nymphal stadia. Other vertebrates like rodents, deer, hedgehogs and birds as well as vegetation characteristics are part of a complex system that influences the interaction between infected ticks and humans. 

Belgium is a Western European country with temperate climate, a warm summer and no dry season [5]. Many vegetated areas in the country provide the environmental conditions to be the habitat of vector and host organisms for PUUV hantavirus and *B. burgdorferi*. The vegetated areas as well as the country’s population density [6] are heterogeneously distributed throughout the country. 

**Figure 1:**
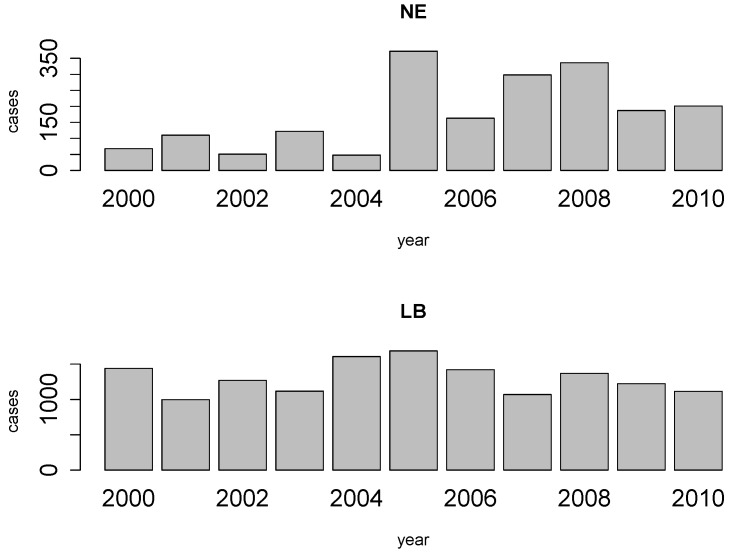
Annual number of cases of NE and LB for the period 2000–2010 in Belgium.

Public awareness and scientific interest concerning LB and NE have risen in recent years as consequence of a notable increase in the number of reported pathological cases and the higher frequency of observed outbreaks [2,7,8,9]. Figure 1, based on reports by the Belgian Scientific Institute of Public Health (IPH) [9], illustrates that the NE and LB reported cases manifest remarkable differences across the temporal dimension and that important outbreaks are observable for both diseases. It will be shown further on that the reported cases are not randomly distributed. While the incidence of NE is larger in the southern part of the country, the number of LB cases is much larger in northern Belgium. 

## 3. Methods

### 3.1. The Gravity Model

GM is the most widely used formulation of spatial interaction analysis [1] and is applied in an extensive range of study fields. The denomination of the GM is owed to its resemblance to Newton’s law of universal gravitation. In its generic form, the GM states that the attraction force *a_ij_* between two entities *i* and *j* is directly proportional to their masses, *m_i_* and *m_j_* and inversely proportional to the squared distance separating them, *d_ij_*, as presented in Equation 1 [10].

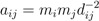
(1)


Mathematically, the GM concept is associated to the concept of entropy maximization as it results from searching the most probable configuration of interaction flows between spatial entities [10,11]. 

Analogies to this concept have been proposed for applications in domains like trade modelling, transportation networks, migration flows, biodiversity monitoring [12], plant diseases [13], *etc*. Several applications are related to human health issues like access to primary health services [14], exposure to pollutants [15] and, in epidemiology, the spread of diseases like measles [16], influenza [17,18], cholera [19] or Hodgkin’s lymphoma [20]. 

The implementation we tested in this study aimed at modelling the disease risk at municipal level, expressed as number of cases per 10,000 inhabitants. The hypothesis behind this implementation is that disease risk is primarily driven by the intensity of interaction between humans and vegetated systems functioning as habitats for NE and LB vectors and hosts. The choice of a spatial interaction model for this analysis, and GM in particular, results from conceiving urban areas and vegetated areas as spatial entities; thereby, acknowledging the importance of the location of these entities with respect to one another. 

The formulation of the GM tested in this study is presented in Equation 2.

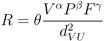
(2)
where *R* is the estimation of disease risk in number of cases per 10,000 inhabitants; *V* is the sum of the surface covered by the vegetated systems in the surroundings of urban areas (*U*); *P* is an estimation of population in *U* and *F* is the fraction of registered companies exerting economic activities implying elevated exposure to the pathogens under study; *d_VU_* is the average distance separating *U* and the surrounding vegetated areas; and *θ, α, β* and *γ* are the model parameters. The variable selection and their implementation in a GM framework rely on current knowledge on NE and LB transmission mechanisms. Some elements are presented hereafter:

1. Several studies have shown the importance of the location of residential areas with respect to forests as risk factor [21,22,23,24,25,26,27,28]. The distance separating urban settlements and forests is also a determinant of the flow of visitors to green areas for recreational purposes [29].
2. The surface covered by vegetative systems is also an important determinant of the magnitude of disease risk and incidence [30]. Besides the ecological effects related to the size and degree of fragmentation of ecosystems, these attributes of vegetated areas affect the kind and intensity of interaction humans have with them. For instance, the size of vegetated areas is an important determinant of the attraction value of green areas [31].
3. Accounting for human activities and their relation to disease risk is a complex matter [32] and no single model element is able to represent this complexity. Nonetheless, it is known that certain occupational groups are highly exposed to tick-bites and/or rodent-borne pathogens. Case-control studies report that foresters, hunters, farmers, amongst other professions, present specially high disease risk as consequence of intensive interaction with the habitat of vector organisms [33,34,35,36]. The connection between occupation and disease risk supports the consideration of the variable *F* in the model as proxy of the exposure to NE and LB pathogens linked to professional activities, as it represents the share of activities like forester, hunter or ranger in the economic structure of the municip

The estimation of the gravity model parameters was conducted on the log-transformed form of Equation 2, as shown in Equation 3 (same symbology as in Equation 2), by using a generalized linear model (GLM). A binomial distribution was used to model the variability of the number of cases in a cohort composed by the population in risk per municipality. This value is expressed as *f(R)* in Equation 3. The link function was the natural logarithm.

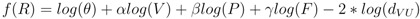
(3)


Opting for GLM to estimate the parameters of Equation 3 implies that the error distribution is not expected to follow a normal distribution, which is likely the case when modelling rate quantities [1]. Details on the estimation of the covariates in Equation 3 are presented in the remainder of this section. Section 3.2 deals with the criteria and methods to estimate the response variable *R*. Section 3.3 and Section 3.4 are devoted to describe the criteria and data sources for estimating the area and location of vegetative systems *V* and population related aspects (*P* and *F* ), respectively. 

### 3.2. Disease Risk Estimator

Epidemiological records provided by the Belgian Institute of Public Health (IPH) were processed in order to compute values of disease risk. These estimations of risk were used as dependent variable when fitting the model. Various disease risk estimators exist, several of them being based on Bayesian statistics. We followed the method proposed by Marshall [37] to compute a local Empirical Bayesian Estimator of risk (EBE). 

The selection of a Marshall’s local estimator to assess risk dynamics in NE and LB is justified by the spatial nature of the triggering factors of disease outbreaks; *i.e*., it is very likely that large variations in terms of risk are present across the country and that neighboring municipalities exhibit similar risk conditions as risk will be greatly influenced by spatially meaningful factors (e.g., landscape aspects, forest characteristics, *etc*.). 

The neighborhood relations among municipalities was established by following a First Order Queen contiguity criterion, *i.e*., two municipalities were considered neighbors when their borders shared at least one point. 

Marshall’s algorithm is represented by the following expression [37]:

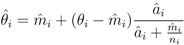
(4)
where 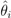 is the EBE for municipality *i, θ_i_* was calculated as the ratio between the number of cases to person-years at risk (*n*) in municipality *i*. 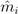 and *â_i_* are the prior mean and variance of relative risk, respectively, calculated over municipalities adjacent to *i*. 

The estimation of person-years at risk *n_i_* in Equation (4) was based on demographic data per municipality concerning the number of inhabitants per age and sex classes obtained from official statistical data sources [6,38] and on the breakdown of reported cases per age and sex class provided by the IPH [9]. The value of *n_i_* resulted from a weighted summation in which weights were assigned in function of the contribution of each sex and age class to the overall number of reported cases. 

EBE values 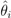 were computed for NE and LB for all municipalities. The estimation of model parameters was done on a sample of 10% of the municipalities. The sample municipalities were chosen such that the whole range of variability of EBE values was represented. The adequacy of the model was tested then against the totality of municipalities. 

### 3.3. Area and Location of Vegetative Systems

Many studies inform on the habitat preferences of the organisms involved in the NE and LB transmission mechanisms. For both diseases the forested areas, and particularly the broad-leaved forests, are the most notorious landscape feature associated to disease risk [30,33]. The set of land cover classes inducing high incidence of LB is larger as more organisms are involved in LB transmission mechanism and ticks are found in a wide range of environment conditions like heathland, coniferous forests, urban green areas, *etc*. [33]. 

Information on location and area of vegetated systems can be extracted from land cover maps or remotely sensed datasets. In this study we evaluated both and generated various vegetation maps that were tested as input in the model. Each of these tests is further referred to as a modeling formulation. 

**Figure 2:**
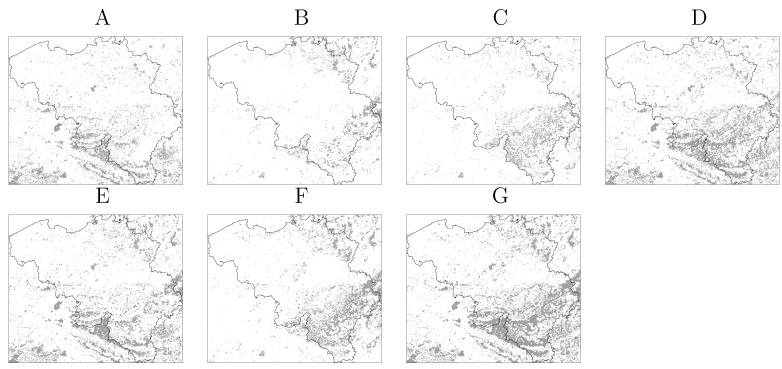
Vegetation maps of Belgium derived from the CORINE land cover map. (**A**) Broad-leaved forest; (**B**) Coniferous forest; (**C**) Mixed forest; (**D**) Broad-leaved and mixed forest; (**E**) Broad-leaved and coniferous forest; (**F**) Mixed and coniferous forest; (**G**) All forest classes. The classes Natural Grasslands, Moors and Heathland and Transitional woodland-shrub are included in all maps. Vegetated area is depicted in gray.

The CORINE land cover (CLC) map [39] was chosen in virtue of its Europe-wide scope in terms of coverage and methodology. The CLC map considers three forest types, broad-leaved, mixed and coniferous, as well as other vegetation classes of relevance for the organisms involved in NE and LB. Each forest type was tested individually as well as combinations of forest types reported to influence NE and/or LB occurrence [30]. In all cases, the vegetation maps included also the following classes: *natural grasslands*, *moors and heath-lands* and *transitional woodland-shrub*, as these may host ticks and rodents as well. This resulted in six binary vegetation maps presented in Figure 2. 

**Figure 3:**
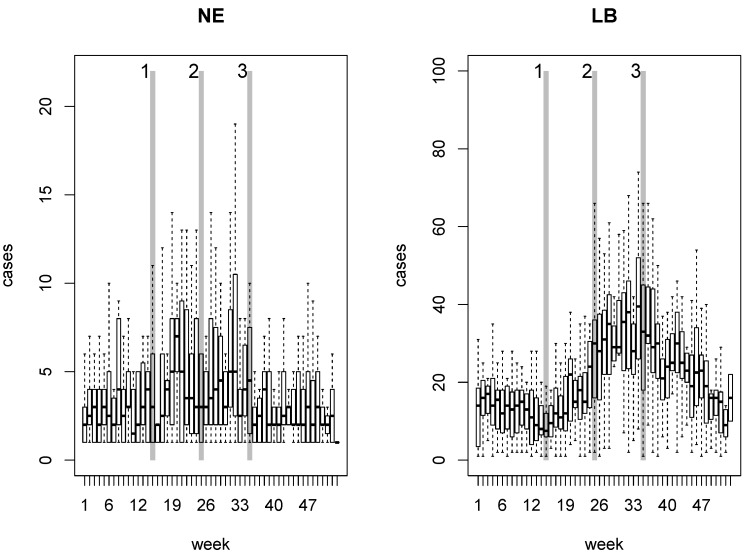
Boxplots of weekly occurrence of NE and LB for the period 2000–2010 in Belgium. The vertical gray lines represent the three dates on which NDVI was computed: Date 1 = week 15, Date 2 = week 25 and Date 3 = week 35.

As for remotely sensed datasets, we utilized the most commonly used vegetation index obtained from space-borne observations: the normalized difference vegetation index (NDVI). The NDVI is computed by normalizing the difference between reflectance values in two regions of the electromagnetic spectrum captured by space-borne sensors and sensitive to vegetation activity. The NDVI is built as:

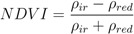
(5)
where *ρ* is the energy reflectance captured by the sensor in the infrared (*ir*) and red (*red*) regions of the electromagnetic spectrum. The data were obtained from measurements by the MODIS sensor on board the TERRA satellite in the year 2005 and delivered in the MOD09Q1 product at a spatial resolution of 250 m [40]. The periodic availability of satellite imagery allowed the calculation of NDVI at three different dates that were defined on the basis of the NE and LB annual occurrence pattern illustrated in Figure 3. 

The three dates indicated in Figure 3 correspond to the weeks 15, 25 and 35 and can roughly be associated to the green-up, climax and declining phases of the vegetation growing season. As illustrated in Figure 3, the first date marks, for both diseases, the beginning of the period of highest occurrence. Around week 25 the highest occurrence of NE has been reached and LB cases show an increasing trend that reaches a maximum around week 35. 

**Figure 4:**
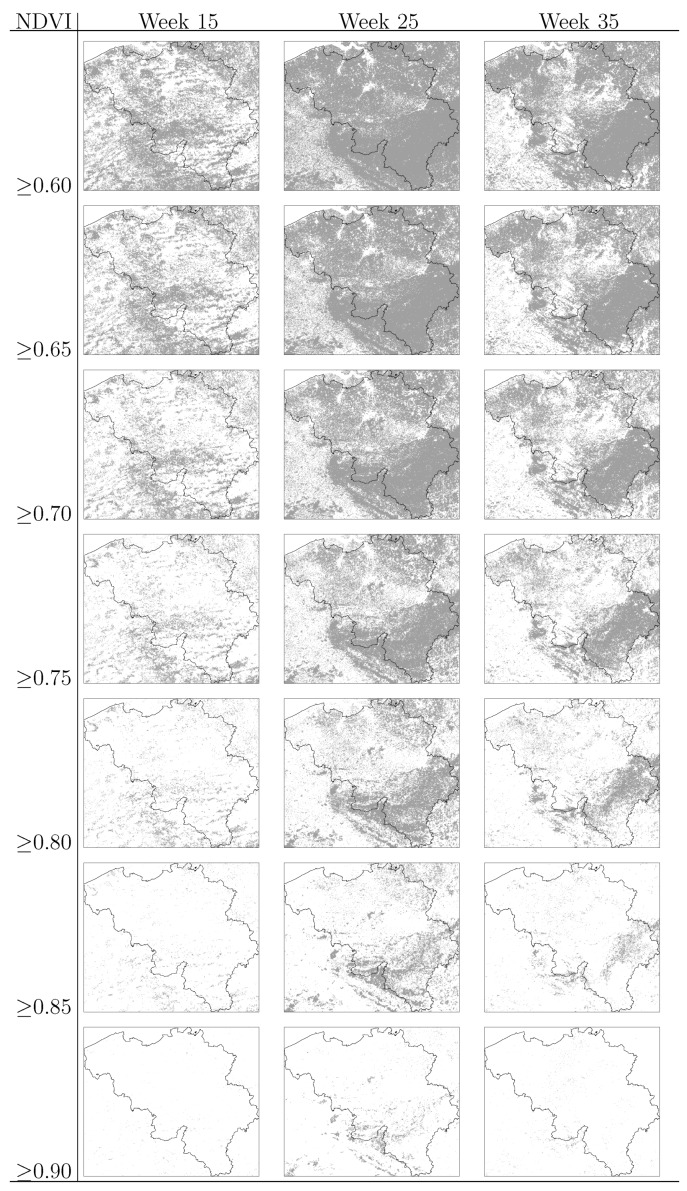
Binary vegetation maps of Belgium based on NDVI critical values at week 15, week 25 and week 35. Areas with NDVI equal or greater than the critical value are depicted in dark gray.

On the basis of the NDVI values, binary maps were produced separating areas below and above a critical value. The evaluated NDVI critical values range from 0.6 to 0.9 representing increasing levels of vegetation greenness and abundance. The binary maps showing areas above and below the critical values are presented in Figure 4. 

### 3.4. Human Population

The location of urban centres was determined using the CLC map; particularly, the classes labelled as *continuous and discontinuous urban fabric* [41]. Mapping the urban areas allowed the computation of Euclidean distances separating these areas and the vegetated areas located within a defined maximum distance. The maximum distance was set to 20 km, as this threshold exceeds the distance reported by previous studies as determinant for attracting visitors to forests [29,42] or for probable contact with disease vectors [22,28]. 

The allocation of population at risk for each urban centre was made by disaggregating the estimated population at risk per municipality (Section 3.2) over the urban centres in proportion to their area size. 

In order to account for the elevated exposure to pathogens associated to certain professional activities [33,34,35,36], the fraction of companies (*F*) devoted to *crop and animal production*, *hunting and related services* and *forestry and logging* was tested as covariate in the model. The value of *F* is meant to be a proxy for exposure related to professional activities at municipal level. It was derived from national statistics per type of economic activity according to the NACE-BEL classification system [43]. 

**Figure 5:**
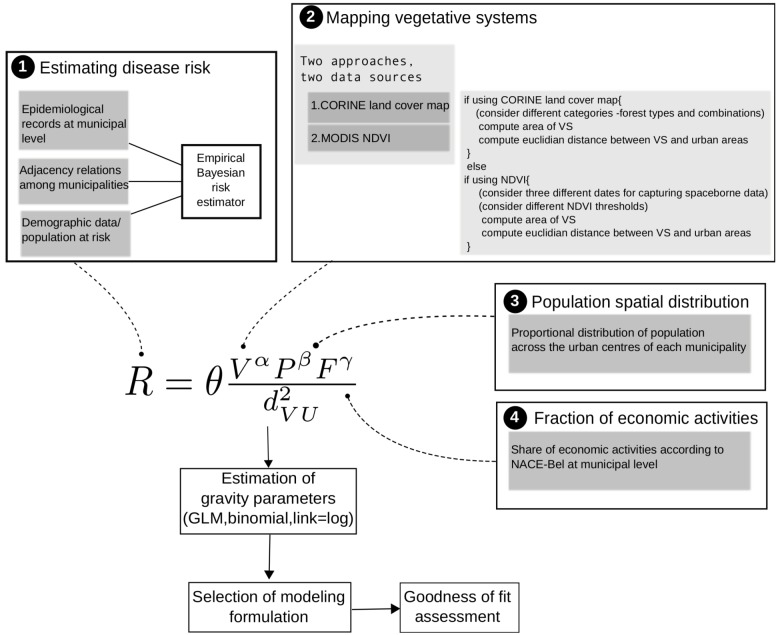
Schematic summary of methods highlighting the four main phases of data acquisition: (1) The estimation of local disease risk; (2) Two approaches to map vegetated systems; (3) The spatial heterogeneity in population density, and; (4) Information on work-related exposure.

The methodological approach presented in the preceding sections is summarized schematically in Figure 5. The four frames shown in Figure 5 represent the major methodological phases. The first frame refers to the derivation of risk patterns from epidemiological records, the adjacency connections among municipalities and the spatial distribution of population. The second frame shows the criteria and data sources used for the generation of vegetation maps, and the third and fourth frames are related to aspects of the human population that may affect disease risk. The last step in the scheme of Figure 5 refers to the goodness of fit assessment in which the different modeling formulations were compared. The comparison criterion was the Akaike information criterion (AIC) [44] and the estimation of spatial autocorrelation of residuals by means of the Moran’s *I* parameter [45]. 

## 4. Results

### 4.1. Disease Risk

**Figure 6:**
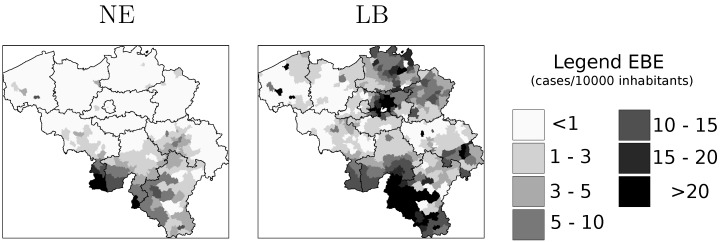
Estimation of disease risk for NE and LB according to Marshall’s local estimator (EBE).

The computation of EBE values revealed heterogeneity in risk grade for both diseases across the country. Figure 6 shows map representation of these values. These maps indicate that for NE, southern Belgium is the most important risk area, especially the region along the Franco-Belgian border. The NE risk grade seems to diminish as one moves in the north-east direction departing from the area of highest risk. The infection risk for LB is spread over a larger part of the country being the most remarkable areas the Walloon region (southern Belgium), including the area where the highest infection risk of NE is located; and northeastern Belgium. The values depicted in Figure 6 were used as reference values when estimating model parameters and when evaluating the models’ goodness of fit. 

**Figure 7:**
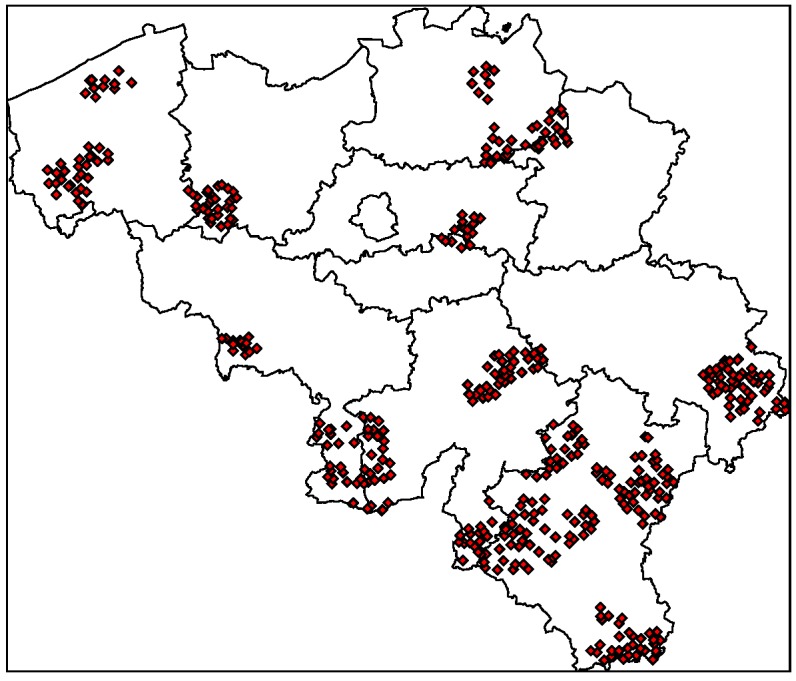
Selected urban centres for fitting the gravity model. Selection was based on the representativeness of the whole range of disease risk grades for both diseases.

The spatial distribution of disease risk shown in Figure 6 was the basis for the selection of municipalities to be sampled. As stated earlier, the main criterion to select municipalities was the representativeness of the whole range of disease risk grades for both diseases. The area covered by the sampled municipalities is presented in the map in Figure 7. 

### 4.2. Model Selection

**Figure 8:**
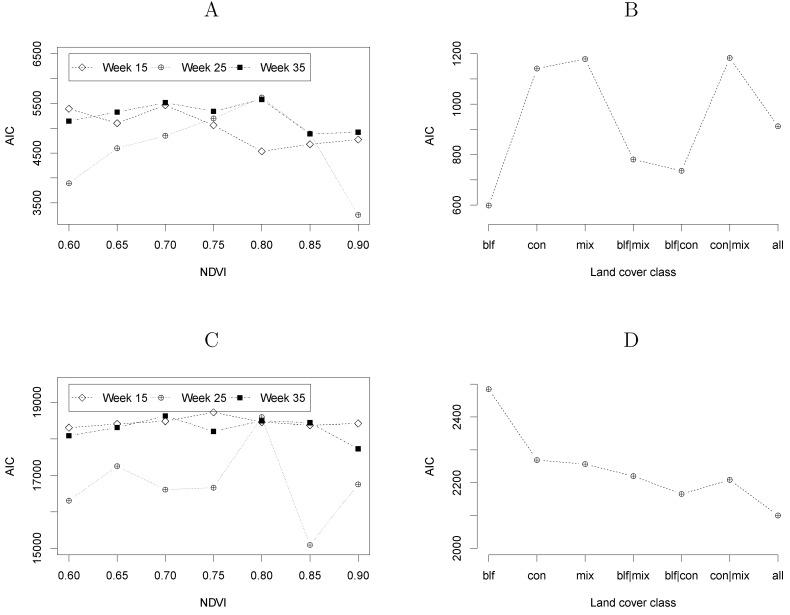
Akaike information criterion (AIC) for each of the tested modelling formulations according to the definition of the vegetated areas. In plots **A** (NE) and C (LB) the data source was MODIS NDVI captured at three different moments of the growing season and applying different values of NDVI as threshold. Plots **B** (NE) and **D** (LB) result from deriving vegetation maps from vegetation classes of the CORINE land cover map.

The plots in Figure 8 show the Akaike information criterion (AIC) for each of the tested modelling formulations. Following the criterion of selecting the lowest AIC values, Figure 8 (A and C) shows that obtaining vegetation maps from NDVI values at week 25 leads to more adequate modelling formulations than NDVI at weeks 15 or 35. Moreover, these plots suggest that the spatial spread of NE and LB can be associated to areas with, respectively, NDVI ≥ 0.9 and NDVI ≥ 0.85 in the period of full vegetation development. 

Figure 8(B and D) shows, for NE and LB respectively, the AIC values of the GM when the vegetation maps were derived from the CLC map. The best modelling formulations were obtained when using the broad-leaved forest class for NE and the combination of all forest classes for LB. These results are in accordance with other studies referring that broad-leaved forests is the most important landscape feature related to NE and that the spatial spread of LB is associated to a larger gamma of land cover classes [30,33]. 

**Figure 9:**
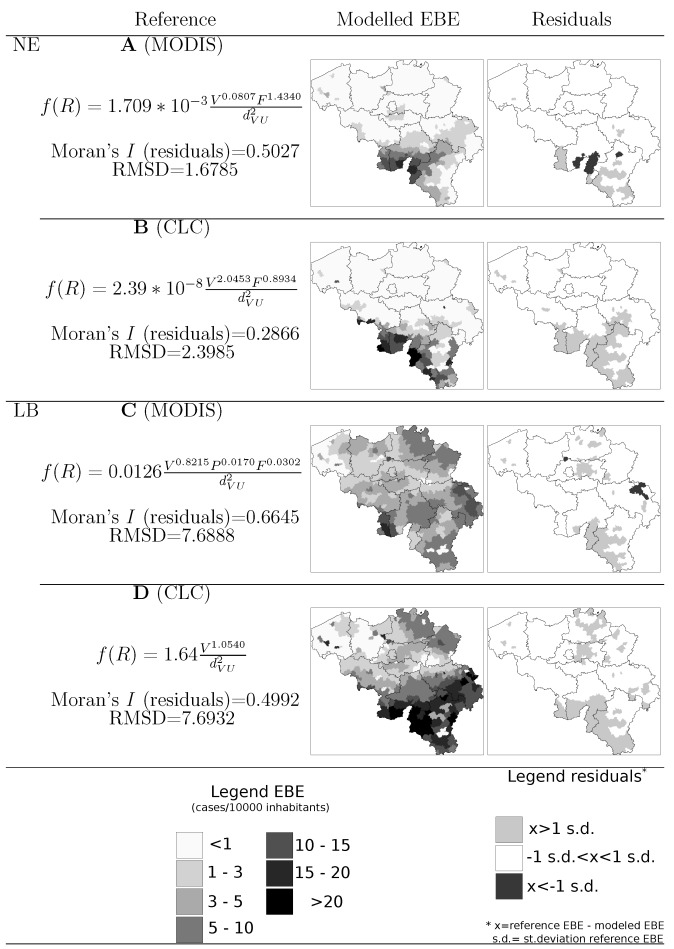
Gravity model equations, residuals Moran’s *I*, root mean squared deviance (RMSD) and map representation of modelled risk estimator and residuals with vegetation maps derived from (A) MODIS NDVI ≥ 0.9 captured at week 25; (B) Broad-leaved forest class from CLC map; (C) MODIS NDVI ≥ 0.85 captured at week 25; (D) All forest classes from CORINE land cover map.

The model formulations with the lowest AIC were selected for further analysis. Non-significant predictors were removed from the models (significance as *ρ* < 0.001) and model parameters were recalculated where applicable. The final GM equations as well as map representations of the modelled EBE values and the model residuals are presented in Figure 9. 

A visual inspection of the maps in Figure 9 suggest the suitability of the models to segment the country into sub-areas in function of disease risk. The most notorious homogeneous risk zones, represented in the reference maps of Figure 6, are identifiable also in the modelled EBE maps and the residuals across the largest part of the country are smaller than 1 standard deviation. The map of residuals also showed that areas with the largest residuals were commonly located in areas of high disease risk, *i.e*., the model succeeded in locating the high risk areas but there, the modelled EBE value is lower than the reference value. 

The root mean squared deviance (RMSD), also indicated in Figure 9, reflected a better fit of the GM for modelling NE with vegetation maps derived from MODIS NDVI as compared with the utilization of the CLC map. In either case, the RMSD is not larger than the standard deviation of EBE values across the country. The Moran’s *I* value indicated a higher degree of spatial autocorrelation in the former model, which means a more clustered pattern in the spatial distribution of residuals. As for LB, the RMSD in both formulations is practically the same and does not exceed the value of standard deviation either. As was the case for NE, the most important difference in the LB model formulation lies in the spatial autocorrelation of residuals. 

## 5. Discussion

The results presented in the previous section verified the adequacy of the concept behind the GM for applications in spatial epidemiology. The suitability of the GM concept is based on the fact that the two diseases under study have a twofold connection with vegetated systems. Firstly, they are the habitat of vectors and other organisms, hence the importance of mapping and measuring vegetation systems. Secondly, human exposure to vector-borne pathogens is greatly determined by the way and intensity of interaction between humans and vegetated systems, and this in turn is shaped by parameters defining the attractiveness of green areas to humans like distance, size, composition, *etc*. 

Our results showed that both land cover maps and space-borne data sources can serve the purpose of locating vegetated systems influencing the spatial spread of disease. In both cases we obtained satisfactory results in the segmentation of areas in function of disease risk grade. As can be observed in the maps of residuals of Figure 9, most of the inaccuracies in the modelled values are underestimations of the actual risk values and are located in areas of epidemiological importance. This can be related to the fact that the reference EBE values are influenced by remarkable outbreaks in both diseases reported in different moments throughout the period 2000–2010. Modelling inter-annual differences in the spatial pattern of risk can be the subject of future research and would favor the use of remote sensing to detect changes in vegetation systems. 

**Figure 10:**
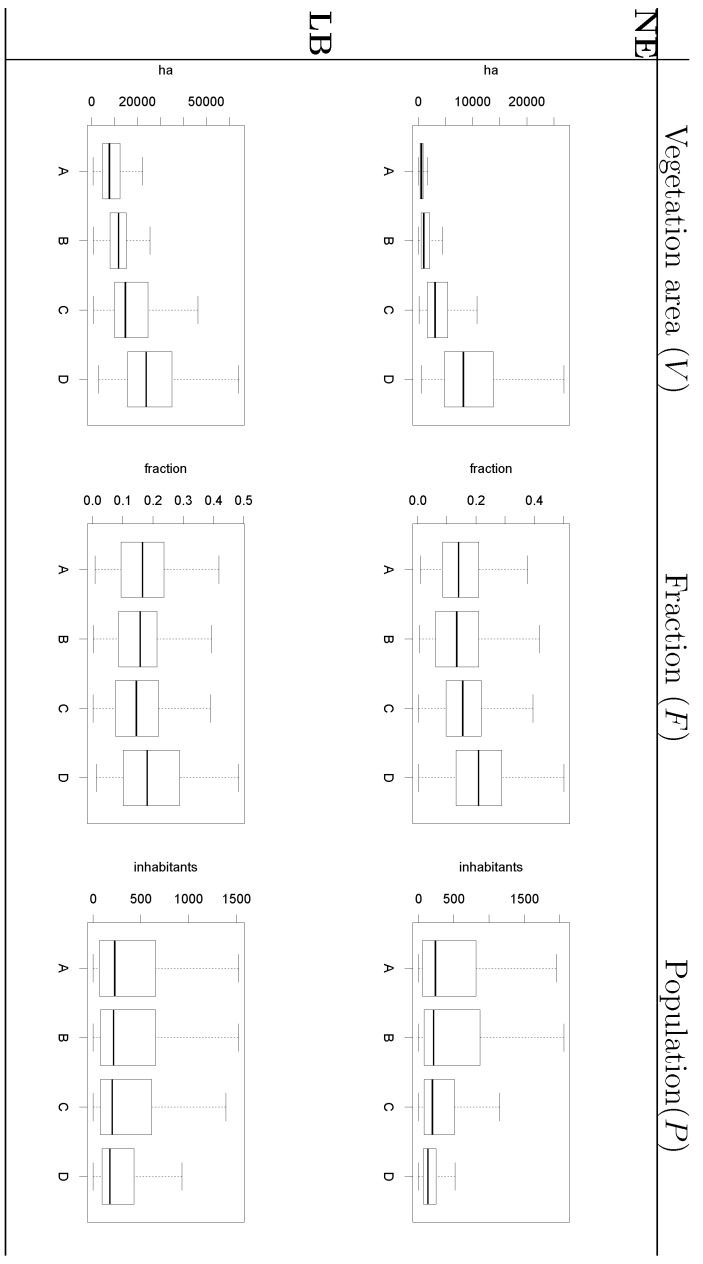
Relation between variables evaluated in the model and disease risk estimator grouped by quartile. Labels A, B, C and D indicate, respectively, the first, second, third and fourth quartile of the local disease risk estimator values.

Figure 10 illustrates the clear connection between the size of vegetated areas and risk grade for both diseases. Figure 10 also shows that municipalities in the quartile of highest risk for NE and LB are those where the fraction of companies exerting forestry-like activities is higher than in the other three quartiles. These facts explain why these two variables were repeatedly found significant in the tested modelling formulations. 

**Figure 11:**
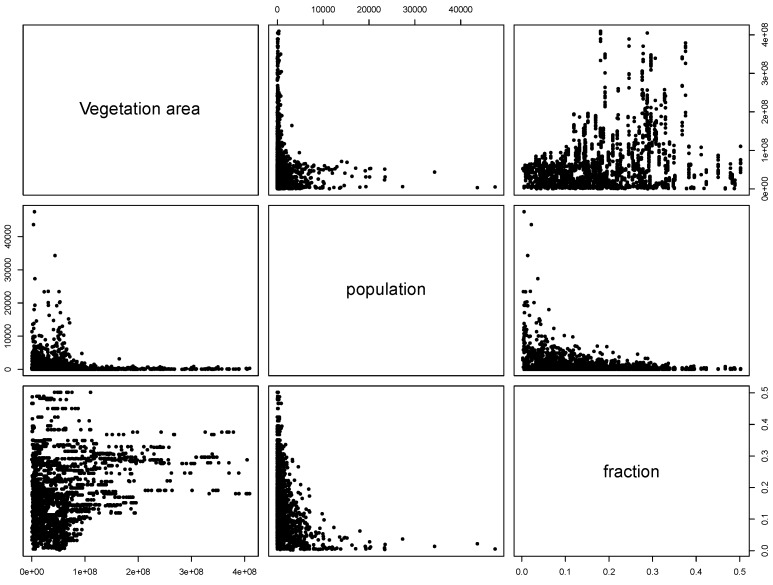
Bivariate relations between tested variables for modelling NE when the vegetation map was derived from MODIS imagery.

Furthermore, Figure 10 also shows that the average population size in the quartile of NE highest risk is lower than in other areas of the country. Despite the correspondence between these variables, *P* was not found a significant predictor of NE. This apparent contradiction is related to the scale of the analysis and the relation between *P* and the other variables in the model, *V* and *F* . Figure 11 shows the bivariate relation between the NE model variables when the vegetation map was derived from MODIS imagery. It follows from the scattering patterns that *P* has some degree of colinearity with variables whose contribution to the model were more significant, *V* and *F* . In contrast, the relation between the variables *V* and *F* does not describe a recognizable pattern, hence the modelled EBE values is the result of the interaction of these variables and the average distance between urban and vegetation entities. This result must not be interpreted as a lack of relevance of the population size as determinant of disease risk rather refers to the impact of the scale of the study. Changing the scale at which the entities are observed can result in different interaction patterns between variables [30]. 

For both diseases the Moran’s *I* value for residuals was higher when vegetation maps were derived from MODIS NDVI. This can be related to inherent differences between a land cover map and vegetation maps defined in terms of NDVI values. In the former case, the vegetation classes are defined in function of the species composition of the vegetation community, whereas in the latter case the leading determinant is vegetation abundance and density, regardless of species composition. Therefore, when using a land cover map, all patches complying with the target cover class are considered in the model even if they are not large and/or dense enough to represent significant disease risk. This can result in a more heterogeneous distribution of residuals across the study area. On the other hand, using land cover maps has the advantage of preventing the confusion between vegetated areas with relevance to disease vectors and other vegetation classes with less importance for the disease under study. 

The two LB models presented in Figure 9 differ in the set of significant covariates composing the GM equations. The equation based on MODIS imagery is defined in terms of *V,P* and *F* whereas the equation obtained when using CLC is built on *V* only. This difference may be explained by a combination of factors. Firstly, zones of elevated LB risk can occur in the context of highly fragmented forest but also in landscapes characterized by large unfragmented forest cover. The finer resolution of CLC map (cell size equal to 100 m) may allow the detection of smaller vegetation patches as compared with MODIS images (cell size equal to 250 m). Thus, the model based on MODIS imagery may need more covariate to reach a high level of accuracy. Secondly, since no distinction is made between vegetation classes when the model is based on MODIS imagery, the application of the NDVI threshold criterion may lead to selecting epidemiologically non-relevant vegetation classes. Additional covariates may then be needed to account for this source of uncertainty. This does not occur when using CLC because one can be certain that forests are the only vegetation class used. 

Finally, it is important to stress that the results on significance of predictors and suitability of data sources may not hold for studies made at other scales. This study aimed at results at country level. Studies focusing on sub-national or regional scales may have more detailed information at disposal, hence the setting of the model may differ. For instance, forest accessibility, management regime or ownership may determine the importance of vegetated areas in human exposure to pathogens together with the attributes considered in this study (size, location, composition). The notion of distance can be refined by incorporating friction factors related to the quality of roads, topography, accessibility, *etc*. Also, research towards the most suited exponent for the distance factor in the GM might result in more accurate treatment of the effect of distance between urban and vegetated areas on disease risk spread. This is beyond the objectives of this study. 

## 6. Conclusions

This study aimed at evaluating GM as an approach to model the spatial spread of VBD, particularly NE and LB in Belgium. The results led us to the following conclusions:

- Information on location, size and composition of vegetated areas is of great importance in modelling the spatial spread of NE, LB and other VBD. Our results show the suitability of both static (land cover maps) and dynamic (space-borne datasets) data sources to derive that information and incorporate it as input of the models.
- Accounting for habitat conditions is of paramount importance when attempting to model VBD. Nevertheless, the models should be enriched by including variables that may come from domains different than ecology or biophysics but that inform on human exposure to pathogens. In the particular case of NE and LB, previous studies highlighted the elevated risk associated to certain occupational groups. Our results showed that data on the number of active companies per branch of the economy at municipal level can provide a significant covariate of risk grade.
- Spatial interaction models have been applied in a large variety of domains where distance and attracting attributes of entities are relevant. The results obtained in this study support the idea of adopting GM or other forms of spatial interaction analysis to model the spread of NE and LB risk and encourage the investigation of the applicability of spatial interaction models in the study of other VBD.
